# Statistical analysis plan for the SOLUTIONS randomised controlled trial with internal pilot: Solution Focused Brief Therapy (SFBT) in 10–17 year olds presenting at police custody

**DOI:** 10.1186/s13063-024-08457-3

**Published:** 2024-09-28

**Authors:** Paul A. Thompson, Gwenllian Moody, Elinor Coulman, Eleri Owen-Jones, Faizan Patel, Kylie M. Gray, Richard P. Hastings, Andrea Longman, Fiona Lugg-Widger, Jeremy Segrott, Julia Badger, Samantha Flynn, Peter E. Langdon, Rebecca Playle

**Affiliations:** 1 School of Education, Language and Communication Sciences, University of Warwick, Coventry, UK; 2 Centre for Trials Research, Cardiff University, Cardiff, UK; 3 Department of Education, University of Oxford, Oxford, UK; 4 Centre for Research in Intellectual and Developmental Disabilities (CIDD), University of Warwick, Warwick, CV4 7AL UK

**Keywords:** Statistical analysis plan, Therapy, Youth offending, RCT

## Abstract

**Background:**

Within England, children and young people entering police custody are referred to Liaison and Diversion (L&D) teams. These teams liaise with healthcare and other support services aiming to divert children and young people away from the criminal justice system. Although targeted psychological interventions are not typically offered to children and young people by L&D teams, evidence suggests that Solution Focused Brief Therapy (SFBT) leads to a reduction in internalising and externalising behaviour problems.

**Methods:**

A two-arm individually randomised controlled trial (RCT) with internal pilot and process evaluation will be conducted with approximately 448 children and young people aged 10–17 years presenting at police custody suites who are referred to the L&D team or recruited via online routes if they have previously presented at any police custody suite in England. The primary outcome is the Self-Report Delinquency Measure (SRDM) at 6 months post-randomisation. Analyses will be performed using intention-to-treat.

**Results:**

The statistical analysis plan (SAP) for the trial is described. The plan details of analyses to be undertaken which will be reported in the primary and any secondary publications. The plan was developed and published prior to locking our database and unblinding to treatment allocation.

**Discussion:**

This RCT will evaluate the effectiveness of SFBT in reducing offending behaviours in CYP presenting at police custody suites including testing of moderating factors and sensitivity of the primary analyses.

**Trial registration:**

ClinicalTrials.gov ISRCTN14195235. Registered on June 16, 2023.

**Supplementary Information:**

The online version contains supplementary material available at 10.1186/s13063-024-08457-3.

## Background

When children are arrested in England many are referred to a Liaison and Diversion (L&D) team. This trial will take place within L&D teams in the area served by Lancashire and South Cumbria NHS Foundation Trust (LSCFT), but also recruit nationally online. The trial aims to test the effectiveness of Solution Focused Brief Therapy (SFBT) as a psychological intervention, with the intention to divert children and young people from serious youth violence and safeguard them from criminal exploitation in the community.

As documented in the published trial protocol [1], the current trial fits with this policy landscape and overall goals as set by NHS England [2]. At the same time, the current project fits with the vision set by the funder of this trial, the Youth Endowment Fund (YEF), which is to prevent children and young people from becoming involved in violence [3].

## Study overview

### Rationale and research question

A systematic review of 38 best evidence studies [4] reported that Solution Focused Brief Therapy (SFBT) led to reductions in internalising and externalising behaviour problems in children and young people. In the proposed research, we will conduct a randomised controlled trial with process evaluation and internal pilot (to assess trial feasibility) to evaluate reduction in offending behaviours where children and young people presenting at a police custody suite will be randomly allocated to receive Solution Focused Brief Therapy (SFBT) plus support as usual (SAU) or SAU alone.

### Objectives

Our primary objective will be to evaluate whether for a sample of 10–17-year-olds presenting at a police custody suite, there is a difference in offending behaviours between participating in the support as usual (SAU) plus Solution Focused Brief Therapy (SFBT) intervention compared to SAU alone, after adjustment for baseline measurements of primary outcome (Self-Report Delinquency Measure; [5]) and stratification variables, verbal IQ and custody suite.

The secondary objectives are to:

- Complete an internal pilot in the first 7 months to examine the feasibility of recruitment and randomisation before continuing with the main trial.
- Generate evidence to consider whether there is a difference between SFBT + SAU and SAU alone on scores for SDQ internalising, externalising and prosocial behaviour outcome measures at 6-month follow-up time point.
- Examine whether there is a difference between SFBT + SAU and SAU alone in offending behaviours, specifically the numbers of arrests, cautions, reprimands, warnings and convictions at 6-month follow-up (adjusted for baseline).
- Examine whether there is a difference between SFBT + SAU and SAU alone on the gang affiliation measure (T-GARM) at the 6-month follow-up.
- Evaluate whether those excluded from school at the point of enrolment or during the trial will have a different rate of offending behaviour than those who have not been excluded.
- Assess the sensitivity of findings under different assumptions with respect to missing data.
- Carry out exploratory subgroup analyses of the primary outcome by learning disability (LD) status, retrospective or in-custody recruitment and callous-unemotional traits.
- Monitor and report adverse events related to SFBT.

### Trial design

The trial is a two-arm individually randomised controlled trial (RCT) of SFBT plus SAU versus SAU alone, involving children and young people (age 10–17 years old) who have presented at one of five police custody suites in the Lancashire and South Cumbria NHS Trust region or who have been recruited nationally via an online advert. The trial involves an internal pilot to be completed at month 12 from the start of the trial, the set-up phase is planned for 5 months and the pilot phase for 7 months. Two hundred eighty-two children and young people participants will be recruited. Although not included in the primary analysis, there is a potential influence of therapist clustering within the intervention arm. This will be investigated as an additional analysis specified in the ‘Additional analyses’ section.

All participants (in both trial arms) will complete assessments at baseline, 6 months post-randomisation and will be given a choice of how these are completed. These assessments can be completed in a number of ways, face-to-face, online on a website, via telephone, via videoconferencing or on paper via the post. Assessment will be monitored throughout the study and a table describing the frequency of assessment method will be presented in the final report.

### Randomisation

Children and young people will be randomised on a 1:1 basis to either the intervention or comparator arm using random permuted blocks, stratified by verbal IQ (dichotomised < 70, ≥ 70) and custody suite. A more detailed description can be found in the randomisation protocol (v0.8 24/05/2022). Random permuted blocks with varying block size (sizes 2, 4, 6) are generated using Stata version 17.0 with the ‘ralloc’ function (senior statistician, Playle generated the lists, so that study statistician, Thompson, remains blind to allocations). The list is then uploaded to the REDCap study database and allocations are automated.

### Sample size

Sample size calculations were conducted using R version 4.1.2 (2021–11-01). Initial sample size estimates were calculated at *n* = 448 assuming 90% power and a conservative correlation between baseline and follow-up, *r* = 0.334 as described in our previously published protocol [1]. We have revisited this sample size calculation in light of new information about the correlation between baseline and follow-up obtained from the START trial using the same outcome measure and in a similar population of adolescents [6]. Similarly, the dropout rates for the START trial showed that our predicted dropout rate (20%) is reasonable given the START trial reported rates of 15% in the intervention arm and 10% in the control arm. We have anticipated further challenges with the accrual rate considering the nature of the participant population, and power was reduced from 90 to 80% power to reduce sample size requirements but still maintain sufficient power for the trial. Our revised sample size estimate is 282 children and young people participants allowing for up to 20% dropout from the trial (*N* = 225). Recruiting this number of children and young people, and on the basis of detecting a minimal clinically important difference (MCID) 0.325 (mean difference of 4 points with SD = 12.32), assuming a correlation between baseline and follow-up of 0.5 [7] and using a two-sided alpha of 0.05, the trial would then be 80% powered.

The sample size has been designed to address the primary analysis only.

### Framework

The trial protocol states that the RCT is designed, ‘to determine whether there is a difference between support as usual (SAU) plus Solution Focused Brief Therapy (SFBT) and SAU alone in reducing offending behaviours in 10–17-year-olds presenting at a police custody suite’. Therefore, the trial is on the basis of superiority of the support with additional therapy arm of the trial.

### Interim analysis

No planned interim analyses. Target sample size will not be recalculated, regardless of rate of recruitment.

### Timing of final analysis

All outcomes will be analysed collectively after the database is locked 1 month following the last 6-month follow-up post-randomisation. One month after completion of baseline data collection and data cleaning, the database will be soft locked to new recruitment and only entry of follow-up data will be permitted. At this point, baseline data summary tables will be generated. After the database is locked after 6-month follow-up post-randomisation, a baseline data table of completers vs non-completers will also be created.

### Timing of outcome assessment

See Table 1.

**Table 1:** Timing of outcome assessments

**Outcomes (secondary)**	**Data collection timepoints**	
	**Baseline**	**6-month follow-up**
Children and young people wellbeing self-report: self-report version of the Strengths and Difficulties Questionnaire	X	X
Children and young people wellbeing parent/ guardian-report: parent-report version of the Strengths and Difficulties Questionnaire	X	X
Gang Affiliation Risk Measure	X	X
MODERATOR: Self-report Callous and Unemotional Traits	X	X
Parent/guardian-report Callous and Unemotional Traits	X	X
MODERATOR: Wechsler Abbreviated Scale of Intelligence (vocabulary and similarities subscales)	X	
Parent/guardian-report other therapies received (including pharmacological)	X	X
Criminal offence data: arrest, caution, reprimands, warnings and conviction data (referrers and the police)	X	X

### Statistical principles

Levels of confidence and *P* values.

All confidence intervals presented will be 95% and two-sided. In addition, all applicable statistical tests will be two-sided and will be performed using a 5% significance level.

#### Adjustment of multiplicity

The overall type I error rate for testing support as usual (SAU) plus Solution Focused Brief Therapy (SFBT) trial arm over the control arm SAU only for the primary endpoint will be controlled at the 2-sided 0.05 significance level. Secondary analyses will control the family-wise error rate using the Holm method.

The Holm method, in a stepwise way, computes the significance levels depending on the *P* value based rank of hypotheses. For the *i*th ordered hypothesis $H(i)$, the specifically adjusted significance level is computed:

$${\alpha }{\prime}\left(i\right)=\frac{\alpha }{m-i+1}$$

where $m$ is the number of hypothesis tests.

The observed *P* value $p(i)$ of hypothesis $H(i)$ is then compared with its corresponding ${\alpha }{\prime}\left(i\right)$ for statistical inference, and each hypothesis will be tested in order from the smallest to largest *P* values$( H(1) , \dots , H(m))$. The comparison will immediately stop when the first ${p\left(i\right)\ge \alpha }{\prime}\left(i\right)$ is observed $(i=1, ..., m)$ and hence all remaining hypotheses of $H(j) (j=i, ... ,m )$ are directly declared non-significant without requiring individual comparison.

### Adherence and protocol deviations

#### Definition and assessment of adherence

SFBT attendance/engagement data will be recorded in logs by practitioners, including start date of CYP engagement with the intervention and number of sessions offered and completed. Six bi-weekly sessions over 12 weeks will be offered and young people should attend all sessions where possible.

The number of sessions delivered will be recorded by practitioners in session summary forms and any implementation challenges recorded.

Adherence is defined as 4 + sessions attended.

#### Presentation of adherence

The number and % of participants for percentage of scheduled sessions attended will be presented in a table. Results will be provided for the treatment group.

#### Definition of protocol deviation

Any deviation from the randomised intervention plan as detailed in the protocol will be considered as a protocol deviation.

#### Presentation of protocol deviation

Prospective, planned deviations or waivers to the protocol will not be allowed (e.g. participants who do not meet the eligibility criteria or restrictions specified in the trial protocol will not be enrolled).

Any accidental protocol deviations will be adequately documented on the relevant forms and reported to the chief investigators immediately.

Deviations from the protocol which occur frequently will be addressed immediately and if appropriate will be classified as a serious breach.

The final analysis will also present the proportions of protocol deviations in a table.

### Analysis population

Children and young people (aged 10–17 years) presenting at custody suites who are referred to a Liaison and Diversion (L&D) team. Participants could be recruited up to 3 months retrospectively to permit capturing full sample requirement. The intention-to-treat population for primary and secondary analyses will include all eligible randomised participants according to the trial arm to which they were randomised irrespective of session attendance. If an ineligible participant is randomised, they will be removed from the dataset and not included in the analysis. The database has several automated eligibility checks before randomisation, so it is unlikely that an ineligible participant will get to the stage of randomisation.

## Study population

### Screening data

The following summaries will be presented for all screened children and young people (overall and by custody suite):

- Enrolment: the number of days recruiting, the number of children and young people screened, the number of children and young people recruited, the number of screened children and young people not recruited, and the reason for non-recruitment.

This information will be included in the CONSORT flow diagram (see Supplementary file—Section A for template).

### Eligibility

Children and young people (aged 10–17 years) will be eligible for this study if they present at a custody suite and are referred to L&D. Children and young people who present with current symptoms of severe mental illness (e.g. psychosis) and are judged to require specialist intervention from child and adolescent mental health will be ineligible.

The number of ineligible patients randomised, if any, will be reported, with reasons for ineligibility. Ineligible patients will be removed from the data and not included into the analysis.

#### Inclusion criteria

- 10–17 years of age
- Referred to the Liaison and Diversion team by the police.

#### Exclusion criteria

- A clinician has judged that the child or young person is presenting with a mental illness of a nature and degree warranting immediate intervention from specialist services, including assessment for detention under the Mental Health Act.
- The young person is to be remanded into custody.
- A child or young person aged 16 years or older judged to lack mental capacity to decide about participating in this trial by staff responsible for gaining informed consent.
- The child or young person is unable to converse in English.
- Parents/guardians are unable to converse in English (at least one must be able to converse in English to complete parent/guardian measures and to provide consent for young people under the age of 16 years).
- Parents/guardians of under 16 s judged to lack mental capacity to decide about participating in this trial by staff responsible for gaining informed consent.

### Recruitment

A CONSORT flow diagram (Supplementary file—Section A) will be used to summarise the number of children and young people who were:

- Assessed for eligibility at screening
- Eligible at screening
- Ineligible at screening*
- Eligible and randomised
- Eligible but not randomised*
- Randomised to each trial arm
- Received the randomised allocation
- Did not receive the randomised allocation*
- Lost to follow-up*
- Discontinued the intervention*
- Randomised and included in the primary analysis
- Randomised and excluded from the primary analysis*

*Reasons will be provided.

### Withdrawal/follow-up

#### Level of withdrawal

The participants’ care will not be affected at any time by declining to participate or withdrawing from the trial because they will still receive services as usual. If a participant initially consents but subsequently withdraws from the trial, clear distinction will be made as to what aspect of the trial the participant is withdrawing from. These aspects will be:

- Withdrawal from intervention (SFBT only)
- Partial withdrawal from future follow-up data collection (e.g. some questionnaires, interviews)
- Withdrawal from previously collected data, prior to data analysis
- Withdrawal of participation in PNC data collection

Participants who withdraw from the trial who have been allocated to receive SFBT will not be able to continue with SFBT as it is unavailable within Liaison and Diversion services outside the context of the trial; they will be able to access usual services only. All participants will be included in the primary analysis unless they withdraw their consent for the use of their data.

#### Timing of withdrawal

Participants have the right to withdraw consent for participation in any aspect of the trial at any time.

#### Reasons for withdrawal

Participants who consent and subsequently withdraw will complete the trial withdrawal form or the withdrawal form will be completed on the participant’s behalf by the site staff/trial team based on information provided by the participant.

#### Presentation of withdrawal/loss to follow-up

The number and % of participants that have withdrawn/loss to follow-up from the study will be presented in a table for all stages. Results will be provided for the treatment group.

### Baseline participant characteristics

#### List of baseline data

Participants will be screened at site and eligibility will be assessed. Potential participant details will be passed from the trial site to the trial team in Warwick. The trial team will contact the participant as per their preferred choice of data collection to take consent and complete the baseline data:

- Age (years)
- Sex/gender
- Who they live with and if they are being looked after
- Whether they are in school
- Type of school
- School year
- Ethnicity
- If they have left school, whether they are in work, an apprenticeship, training, the armed forces or unemployed.
- If English is their first language
- GP contact details
- Medications and treatments (including talking therapies that are being received), collected at baseline and 6 months
- Baseline outcome measures completed (WASI-II is to be completed with researcher assistance [telephone, teleconferencing or face-to-face])
  1. Children and young people wellbeing self-report: self-report version of the Strengths and Difficulties Questionnaire
  2. Children and young people wellbeing parent/guardian-report: parent-report version of the Strengths and Difficulties Questionnaire
  3. Gang Affiliation Risk Measure
  4. Self-report Callous and Unemotional Traits
  5. Parent/guardian-report Callous and Unemotional Traits
  6. LD: Wechsler Abbreviated Scale of Intelligence (vocabulary and similarities subscales)
  7. Parent/guardian-report other therapies received (including pharmacological)
  8. Criminal offence data: arrest, caution, reprimands, warnings and conviction data (referrers and the police) from the preceding 6 months

#### Descriptive statistics

Characteristics of each trial arm group will be summarised descriptively, both as randomised and as analysed in the primary analysis (Supplementary file—Table B1).

Categorical data will be summarised by numbers and percentages. Continuous data that follow a normal distribution will be summarised using means and standard deviations while skewed continuous variables will be summarised using medians and interquartile ranges. Histograms and boxplots will be used to check the distribution and possible outliers for continuous variables. Tests of statistical significance will not be undertaken for baseline characteristics [8]; rather the clinical importance of any imbalance will be noted. Parent/guardians will respond to two check lists of seven items each, medications and therapies. The data will relate to process evaluation and will be reported descriptively and with summary statistics to inform description of the population.

## Analysis

### Outcome definitions

#### Primary outcome(s)

The primary outcome measure for this trial is the Self-Report Delinquency Measure at 6 months post-randomisation (SRDM; 9).

#### Timing, units and derivation of primary

Primary outcome is collected at baseline and 6 months post-randomisation. The SRDM is a derived total score following Smith and McVie [9] and the units are a relative measure of delinquency. The SDRM is a measure comprising 15 items pertaining to antisocial behaviours (e.g. burglary, violence). It requires children and young people to respond with yes or no with reference to a time period (6 months). They then report the estimated frequency of the behaviour, and whether they have ever been caught. Each items frequency is scored 0–5, 6–10 is scored 6 and 11 + is scored 11. Minimum score would be 0 and maximum number of delinquent behaviours would be 165 (15 × 11). On this basis, we may have a skewed continuous distribution, so a log transformation may be required after inspection of model residuals. In addition, there may be a number of individuals where this is their first time in a custody unit, so there is a possibility of floor effects depending on the frequency of their delinquent behaviour. A higher number of delinquent behaviours is bad, so a reduction in the outcome indicates an effective treatment. Baseline and 6-month follow-up data will only be used in the primary analysis.

#### List of secondary outcomes

Secondary participant reported outcome measures include:

- Criminal offence data for participants during the previous 6-month time period (data held in the Police National Computer). We aim to initially collect crime data over the 6-month period prior to the randomisation, at the 6-month follow-up. We aim to analyse the following counts individually:
  - Number of arrests
  - Number of cautions
  - Number of reprimands
  - Number of warnings
  - Number of convictions
- Emotional and behavioural difficulties: the parent/guardian and self-report versions of the Strengths and Difficulties Questionnaire (SDQ) will be used to assess children and young people well-being (including internalising, externalising and prosocial behaviours). The SDQ is a robust and well-validated measure of behavioural and emotional problems [10], measured over the preceding 6 months. The following subscales will be analysed individually for both parent/guardian and self-report:
  - Internalising problems
  - Externalising problems
  - Prosocial behaviour
- Gang affiliation: The Gang Affiliation Risk Measure (T-GARM; [11, 12]) is a 15-item measure of gang affiliation that was developed with teenagers.

#### Order of testing

Secondary participant reported outcomes are tested in the order listed in ‘List of secondary outcomes’ section.

#### Timing, units and derivation of secondaries

Secondary outcomes are generally collected at baseline and 6 months post-randomisation.

- Criminal offence data: We aim to initially collect crime data over the 6-month period prior to the commencement of treatment, at the 6-month follow-up. Baseline and 6-month follow-up data will be collected and used in secondary analysis. For each measure, a count will be recorded.
- Emotional and behavioural difficulties: Baseline and 6-month follow-up data will only be used in the secondary analysis. The SDQ consists of 25 items which are each scored on a 3-point Likert scale (0, 1, 2). Three subscales will be used: (i) externalising problems—ranges from 0 to 20 and is generated by summing the scores of the conduct and hyperactivity subscales; (ii) internalising problems—ranges from 0 to 20 and is generated by summing the emotional and peer problems subscales; and (iii) prosocial behaviour—ranges from 0 to 10 and is generated by summing prosocial behaviour items. Total scores for the subscales can be generated if no more than three items are missing; otherwise, a missing value is generated for the subscore.
- Gang affiliation: Baseline and 6-month follow-up data will only be collected and used in secondary analysis. There are 15 binary (yes/no) items that are summed giving a range 0–15 total score. The score will be analysed as a continuous measure but to aid interpretation, a total score of 7 or more would indicate risk of gang affiliation and would suggest early intervention support is provided. The measure developers provide no guidance on item level missingness or scoring with missingness. Strategies for dealing with missing data are detailed in ‘Missing data in item level data’ section for this measure.

## Analysis methods

### List of methods and presentation

#### Internal pilot study

Statistical analysis for internal pilot feasibility outcomes will be primarily descriptive. Feasibility outcomes (primary outcome measures and all secondary measures) will be estimated as frequencies and percentages, means and standard deviations, or medians and interquartile ranges as appropriate. Feasibility outcomes will be assessed against the pre-specified progression criteria (Supplementary file—Section C).

#### Primary outcome analysis

Our primary outcome analysis will include all randomised participants who provide outcome data (i.e. a modified intention to treat analysis set) and compare mean scores between arms on the SRDM at 12 months post-randomisation using linear regression, adjusting for baseline SRDM score, verbal IQ, sex, age and custody suite (include ‘online’) to investigate the overall effect of the intervention on post-randomisation measures.

$${Y}_{i}={\beta }_{0}+{\beta }_{1}{SDR{M}_{BL}}_{i}+{\beta }_{2}{TX}_{i}+{\beta }_{3}{VIQ}_{i}+{\beta }_{4}{Age}_{i}+{\beta }_{5}{Sex}_{i}++ {\varepsilon }_{i}$$

$${\varepsilon }_{i}\sim N\left(0,{\sigma }^{2}\right)$$

where ${Y}_{i}$ are the SDRM scores; $SDR{M}_{BL}$ are the baseline SDRM scores; $TX$ is the treatment/control variable indicator; *Custody* is the indicator of custody suite (strata, 6 levels: Blackburn, Preston, Blackpool, Harrow, Burnley and online); *VIQ* is the verbal IQ of the children and young people (binary; ≤ 70 or > 70) [13]; *Age* is the continuous measure of age in years; *Sex* is the biological sex indicator of the adolescent; and ${\varepsilon }_{i}$ is the individual level variation. Custody suite has been introduced into the model as a fixed effect as it is a stratifying variable in the randomisation [14].

We will use simple coding for the contrast of custody suite, so that our intercept retains the grand mean and nominally use ‘online’ as our reference level.

Distributional assumptions for the primary linear model will be checked and alternative methods are listed in ‘Alternative methods if distributional assumptions not met’ section. Model output will be reported using Supplementary file—Table B3.

#### Secondary outcome analysis

The SDQ for both parent-report and self-report versions (analysed separately) and the T-GARM will be analysed following the same method as the primary outcome. The distributions of these secondary outcomes will be assessed prior to conducting the analysis. If skew is significant and residuals assumptions are not met, then a Poisson or negative binomial model will be specified (see below, under count variables). If range restriction is apparent (significant floor and ceiling effects in distribution plots), then we will use a Tobit regression [15, 16], as follows:

$${Y}_{i}|{U}_{1j}={\beta }_{0}+{\beta }_{1}{GARM}_{i}+{\beta }_{2}{TX}_{i}+{\beta }_{3}{VIQ}_{i}+{\beta }_{4}{Age}_{i}+{\beta }_{5}{Sex}_{i}+{\varepsilon }_{i}$$

$${\varepsilon }_{i}\sim N\left(0,{\sigma }^{2}\right)$$

$${\mathrm Y}_{\mathrm i}=\left\{\begin{array}{l}\mathrm a\;\mathrm Y_{\mathrm i}^\ast\;\mathrm{if}\;\mathrm Y_{\mathrm i}^\ast\leq\mathrm l\\\mathrm Y_{\mathrm i}^\ast\;\mathrm{if}\;\mathrm l<\mathrm Y_{\mathrm i}^\ast<\mathrm r\\\mathrm b\;\mathrm Y_{\mathrm i}^\ast\;\mathrm{if}\;\mathrm Y_{\mathrm i}^\ast\geq\mathrm r\end{array}\right.$$

where $l$ and $r$ are the left and right censoring thresholds respectively. ${Y}_{i}^{*}$ is considered to be a latent partially observed variable that is able to take values beyond the thresholds.

Remaining secondary outcomes, number of criminal offences (arrests, cautions, reprimands, warnings and convictions), will be analysed similarly but use generalised linear model given that these are counts. For count variables, we will use a Poisson (or negative binomial, as necessary) model checking for zero inflation and overdispersion, as follows:

$${g(Y}_{i})={\beta }_{0}+{\beta }_{1}{BL}_{i}+{\beta }_{2}{TX}_{i}+{\beta }_{3}{VIQ}_{i}+{\beta }_{4}{Age}_{i}+{\beta }_{5}{Sex}_{i}+{\varepsilon }_{i}$$

$${\varepsilon }_{i}\sim N\left(0,{\sigma }^{2}\right)$$

Note: $g\left(.\right)={log}_{e}(.)$, where $g\left(.\right)$ is the log link function for the secondary outcome measures, whereas the primary outcome, $g\left({Y}_{i}\right)={Y}_{i}$. *BL* is the baseline number of offences. Model outputs will be reported using Supplementary file—Table B6.

Effect sizes will be calculated based on the adjusted mean difference between the SAU plus intervention and SAU alone group (controlling for baseline) using the formula [17]:

$$Hedge{s}{\prime}g=\frac{{M}_{1}-{M}_{2}}{{SD}_{pooled}}$$

$${SD}_{pooled}=\sqrt{\frac{({SD}_{1}^{2}+{SD}_{1}^{2})}{2}}$$

The effect size will also be reported with 95% confidence intervals defined:

$$g\pm {\Phi }^{-1}(1-\left(\alpha /2\right)){g}_{se}$$

where ${\Phi }^{-1}$ is the percent point function of the normal distribution, and ${g}_{se}$ is the standard error of the *g* statistic.

$${g}_{se}=\sqrt{\frac{{n}_{1}+{n}_{2}}{{n}_{1}{n}_{2}}+\frac{{g}^{2}}{2({n}_{1}+{n}_{2})}}$$

All parameter estimates from the models will be reported with 95% confidence intervals.

Effect sizes from count models will report rate ratios derived by exponentiating the parameter estimates.

For the remaining secondary outcomes, their effect sizes will be reported as either Hedges’ *g* (Tobit, same as primary outcome) or rate ratios (all other secondary outcomes, exponentiated parameter estimates), given that generalised linear models with log link function are used to model the data and that the measures are positively scored integers with some amount of skew anticipated [18].

#### Covariate adjustment

We will assess any imbalance of baseline covariates for possible inclusion in the primary analysis model where large imbalances are noted. However, due to the sample size, we do not anticipate substantial issues in this respect.

If sufficient data is available, for the PNC data secondary outcomes (criminal offence data), we will adjust the corresponding secondary analysis model for a dummy indicator of school exclusion. This addresses secondary object point 6: Evaluate whether those excluded from school at the point of enrolment or during the trial will have a different rate of offending behaviour than those who have not been excluded.

#### Assumption checking

1. Linearity—plotting residuals vs predictor(s). If a structure is present, then transformation or an alternate model specification is required (i.e. GLM).
2. Homogeneity of variance—variance of the residuals across groups is the same. There is scope to fit models allowing for heterogeneous groups, but the setup is different (generalised linear mixed model—GLMM).
3. Residuals are approximately normally distributed—plotting QQ plot.

#### Alternative methods if distributional assumptions not met

If distributional assumptions are not satisfied, as appropriate, a generalised linear mixed model with alternate link function will be used.

The distributions of the primary outcomes will be assessed prior to conducting the analysis, if variables are skewed, then a Poisson model will be specified, as follows:

$${g(Y}_{i})={\beta }_{0}+{\beta }_{1}{BL}_{i}+{\beta }_{2}{TX}_{i}+{\beta }_{3}{VIQ}_{i}+{\beta }_{4}{Age}_{i}+{\beta }_{5}{Sex}_{i}+{\varepsilon }_{i}$$

$${\varepsilon }_{i}\sim N\left(0,{\sigma }^{2}\right)$$

Note: $g\left(.\right)={log}_{e}(.)$, where $g\left(.\right)$ is the log link function for the primary outcome measure.

Alternatively, data transformation could be used but use of the GLMM is preferable.

#### Sensitivity analyses

Two types of sensitivity analysis will be conducted:

- Exploring the impact of missing data on trial outcomes by investigating likely missing data mechanisms and re-fitting the primary outcome within a multiple imputation framework (including exploring MAR and MNAR mechanisms via delta-based controlled multiple imputation). Imputation variables for the model will include all covariates and the outcome appearing in the analysis as per recommendation by White et al. [19]. In addition, variables that are predictive of missingness are included on the basis of strength of association with response variables. Also, any variables that explain response or non-response [20].

We will summarise the extent of missing data in all outcomes and their respective control variables (Supplementary file—Tables B2 and B7). A full multiple imputation strategy will be used if more than 5% of data in the primary model is missing. Alternatively, we will impute if more than 10% of data for a single variable is missing. We will use the multiple imputation by chained equations approach via the *mice* package in R [21] and generate at least 10 imputed datasets, but will be proportionate to the percentage of missingness (i.e. larger proportions will have more imputed data sets generated). We will then estimate the intervention effect for each imputed dataset and pool the results using Rubin’s combination rules for standard errors.

##### Missing data in item level data

The primary outcome measure’s total score will be imputed directly. For secondary outcomes, we will not impute the PNC data as this will be assumed to be complete and counts of offences will not be imputed. Total scores of the SDQ will be imputed directly given that specific scoring rules for item level missingness are provided by the developers. For the GARM measure, any missing item level data will be imputed using the chained equation approach, and imputed items summed for each imputed dataset to get total score per imputed dataset. Each item’s imputation model will use other items and covariates specified in the analysis model as predictors.

Following creation of the imputed datasets, the corresponding total scores will be calculated using the imputed item level data. All imputed datasets will then fit the primary and secondary models and pool estimates following Rubin’s rules.

##### Primary outcome

Given that each item is a count, we will use a Poisson regression (or negative binomial, if over dispersed) within the imputation model for each item.

##### Secondary outcome

Similarly, the correct link function will be used according to the item’s structure for each of the secondary outcomes, i.e. binary or categorical accordingly. Therefore, a logistic or ordinal model will be used in the imputation for these items.

- Exploring the impact of different levels of intervention receipt on outcomes. We will use two-stage least squares instrumental variables (IV) regression to examine the effect of the intervention in those who receive varying levels of it. The proportion of sessions attended out of a maximum of six will be the instrumental variable in this analysis. The control group attendance will be set to zero and those intervention group will be assigned the number of sessions attended for the IV regression analysis (Supplementary file—Table B8).

Adherence will be categorised for the purposes of summary tabulation: attendance of ≥ 4 sessions (max number of sessions offered = 6).

Fidelity will be calculated as the average session score averaged across session to generate a single fidelity score. Fidelity items will be scored 0, 0.5 and 1. Total fidelity session score will be out of 18 or 20 depending on time point.

Both fidelity and adherence analyses will use a two-stage least square approach to estimate the model and Huber-White standard errors reported which are robust to clustering. The R packages ‘ivpack’ and ‘ivreg’ will be used to implement the two-stage instrumental variable analysis [22, 23]. Compliance (session adherence, i.e. number of sessions) will be instrumented by the intervention allocation [24]. The stage 1 model is defined as follows:

$${Compliance}_{k}={\beta }_{0}+{\beta }_{1}{TX}_{k}+{\varepsilon }_{jk}$$

Predicted values for ${Compliance}_{k}$ from the stage 1 model will be included in the stage 2 model, as follows:

$${Y}_{ik}={\beta }_{0}+{\beta }_{1}{\widehat{compliance}}_{k}+{\beta }_{2}{baseline}_{ik}+{\beta }_{3}{Custody}_{k}+{\beta }_{3}{VIQ}_{k}+{r}_{ik}$$

#### Subgroup analyses

In addition to the primary and secondary outcomes, we have considered that the following outcomes may moderate the outcomes of this trial (Supplementary Table B4):

- Callous and unemotional traits: This will be measured, at baseline and 6-month follow-up using the 24-item Inventory of Callous and Unemotional Traits—Parent/guardian Report and Youth Self-Report versions [25] which are robust and well validated instruments [26]. We will fit two moderation models for this variable to investigate the effect of moderation of treatment outcomes, but change may also occur as a consequence of treatment, so fitting both models permits us to disentangle these effects.
- Learning disabilities (LD): Children and young people will be invited to complete two subtests of the Wechsler Abbreviated Scale of Intelligence-II (WASI-II; 25) to index their verbal IQ. This scale is to be administered with a researcher (face-to-face, telephone, videoconferencing). The two subsets are to be included are vocabulary (31 items) and similarities (24 items). Raw scores are converted to scaled scores and summed, these are then age adjusted and a standardised score is created. The standardised score will be used in the moderation analysis.
- Retrospective or in-custody recruitment: This is a variable indicating whether the participant was recruited while in the custody suite or whether they were recruited retrospectively to the study within the 3-month window.

A moderation analysis will adjust the primary analysis with the inclusion of the moderator as a main effect and interaction between moderator and randomised group indicator. For example, the learning disabilities moderator analysis is as follows:

$${Y}_{i}={\beta }_{0}+{\beta }_{1}{SDR{M}_{BL}}_{i}+{\beta }_{2}{TX}_{i}+{\beta }_{3}{VIQ}_{i}+{\beta }_{4}{Age}_{i}+{\beta }_{5}{Sex}_{i}+{\beta }_{6}{LD}_{i}+{\beta }_{7}{TX}_{i}*{LD}_{i}+{\varepsilon }_{i}$$

$${\varepsilon }_{i}\sim N\left(0,{\sigma }^{2}\right)$$

where, ${Y}_{i}$ are the SDRM scores; $SDR{M}_{BL}$ are the baseline SDRM scores; $TX$ is the treatment/control variable indicator; *Custody* is the indicator of custody suite (strata, 6 levels: Blackburn, Preston, Blackpool, Barrow, Lancaster and online. These will be included as fixed effects rather than a random intercept); *VIQ* is the verbal IQ of the children and young people (binary; ≤ 70 or > 70); *Age* is the continuous measure of age in years; *Sex* is the biological sex indicator of the adolescent; ${LD}_{i}$ is learning disability status; ${TX}_{i}*{LD}_{i}$ is the interaction of learning disability status and treatment/control indicator; and ${\varepsilon }_{i}$ is the individual level variation.

### Additional analyses

#### Clustering via multilevel model

We will additionally consider the role of therapists as a source of clustering. As therapists will deliver the intervention to individuals allocated to the intervention arm only, this will be a form of partial nesting and may lead to an underestimation of standard errors (and thus inflated type I error) if not appropriately accounted for. We will also report intra-cluster correlation coefficients, the number of clusters and cluster sizes. To account for any clustering, we will fit a heteroscedastic partially nested mixed-effects model structure [27]. The model will have a three-level structure, level 1 (individual) and level 3 (therapist). Verbal IQ, age, sex and intervention variables will be included at level 1 and custody suite at level 2 (Supplementary Table B5).

$${Y}_{ij}={\beta }_{0j}+{\beta }_{1j}{SDR{M}_{BL}}_{ij}+{\beta }_{2j}{TX}_{ijk}+{\beta }_{3j}{VIQ}_{ij}+{\beta }_{4j}{Age}_{ij}+{\beta }_{5j}{Sex}_{ij}+{U}_{j}{TX}_{ij}+{r}_{ij}(1-{TX}_{ij})+{\varepsilon }_{ij}{TX}_{ij}$$

$${\varepsilon }_{ijk}\sim N\left(0,{\sigma }^{2}\right)$$

$${U}_{j}\sim N\left(0,{{\sigma }^{2}}_{u}\right)$$

$${r}_{ij}\sim N\left(0,{{\sigma }^{2}}_{r}\right)$$

where ${Y}_{ij}$ are the SDRM scores; $SDR{M}_{BL}$ are the baseline SDRM scores; $TX$ is the treatment/control variable indicator; *Custody* is the indicator of custody suite (strata, 6 levels: Blackburn, Preston, Blackpool, Barrow, Lancaster and online. These will be included as fixed effects rather than a random intercept); *VIQ* is the verbal IQ of the children and young people (binary; ≤ 70 or > 70); *Age* is the continuous measure of age in years; *Sex* is the biological sex indicator of the adolescent; ${r}_{ij}$ is the individual level variation in the non-clustered control arm; ${\varepsilon }_{ij}$ is the individual level variation in the clustered arm; and ${U}_{j}$ is the random intercept term for therapists.

In the first instance, we will assume compound symmetry as our correlation structure, but will investigate the autocorrelation plot and adjust the correlation structure as necessary, for example first-order autoregressive (AR1) residuals.

Initially ICCs, at therapist level, will be calculated for the null model (without covariates predicting the SDRM), and then for the primary model (i.e. the model including the baseline SDRM score, age, sex, verbal IQ and custody suite as covariates).

#### Longitudinal follow-up analyses

We will fit linear mixed models, accounting for repeated post-randomisation measures, SRDM outcome (6 and 12 months post-randomisation) within participants, adjusting for baseline measures, custody suite and counsellors to investigate the overall effect of the intervention on post-randomisation measures.

$${Y}_{ijk}={\beta }_{0jk}+{\beta }_{1jk}{SDR{M}_{BL}}_{ijk}+{\beta }_{2jk}{TX}_{ijk}+{\beta }_{3jk}{VIQ}_{ijk}+{\beta }_{4jk}{Age}_{ijk}+{\beta }_{5jk}{Sex}_{ijk}+{U}_{0k}+{U}_{1k}{time}_{ij}+{\varepsilon }_{ijk}$$

$${\varepsilon }_{i}\sim N\left(0,{\sigma }^{2}\right)$$

$${U}_{0j}\sim N\left(0,{{\sigma }^{2}}_{u2}\right)$$

$${U}_{1j}\sim N\left(0,{{\sigma }^{2}}_{u1}\right)$$

In this case, the effect size and 95% confidence interval will be calculated using given as Hedges *g* [17] for cluster randomised designed analysed via multilevel models and allowing for unequal cluster sizes. According to the two-level LMM for primary outcome, a sample estimate of the effect size equivalent to Hedges’ *g* with 95% confidence interval is defined as:

$${\widehat{\Delta }}_{g}=\frac{\widehat{{\beta }_{1}}}{{S}_{T}}\sqrt{1-\frac{2\left(n-1\right)\rho }{N-2}}$$

where ($\widehat{{\beta }_{1}}$) is the adjusted mean difference in SRDM score between trial arms; ${S}_{T}$ is the within group pooled standard deviation (unconditional sample variance).

$${S}_{T}^{2}=\frac{{\sum }_{i=1}^{{m}^{I}}{\sum }_{j=1}^{{n}_{i}^{I}}{({Y}_{ij}^{I}-{Y}_{i..}^{I})}^{2}+{\sum }_{i=1}^{{m}^{C}}{\sum }_{j=1}^{{n}_{i}^{C}}{({Y}_{ij}^{C}-{Y}_{i..}^{C})}^{2}}{N-2}$$

where ‘*mI*’ is the total number of counsellors in the intervention sample, and ‘*n*^*I*^’ the total number of participants (equivalent definitions apply for the control group, but with the ‘*C*’ designation). ${Y}_{i..}^{I}$ and ${Y}_{i..}^{C}$ are the mean outcomes among intervention and control counsellors respectively.

The remaining part of the ${\widehat{\Delta }}_{g}$ equation makes the adjustment for clustering. The two intra-class correlation coefficients at the counsellor ($\rho$) level are defined as follows:

$$\rho =\frac{{\sigma }_{B}^{2}}{{\sigma }_{B}^{2}+{\sigma }_{W}^{2}}=\frac{{\sigma }_{B}^{2}}{{\sigma }_{T}^{2}},$$

where ${\sigma }_{B}^{2}$ is the between-counsellor variance, and ${\sigma }_{W}^{2}$ is the within-counsellor variance.

For the remaining secondary outcomes, their effect sizes will be reported as either Hedges’ *g* (Tobit, single level model and same as primary outcome) or rate ratios (all other secondary outcomes, exponentiated parameter estimates), given that generalised linear mixed effects models with log link function are used to model the data and that the measures are positively scored integers with some amount of skew anticipated [18].

### Harms

The number (and percentage) of patients experiencing each AE/SAE will be presented for each trial arm categorised by severity. For each patient, only the maximum severity experienced of each type of AE will be displayed. The number (and percentage) of occurrences of each AE/SAE will also be presented for each trial arm. No formal statistical testing will be undertaken.

### Statistical software

All statistical analyses will use R version 4.1.2 (2021–11-01) with additional packages: tidyverse, VGAM, lme4, lmerTest, performance, mice, psych, ivreg and ivpack. Tobit models with random effects will be fitted using Stata 17 using the metobit function.

## Supplementary Information

Supplementary Material 1.

Supplementary Material 2.

## Data Availability

Due to the nature of the research, due to the sensitive and personal nature of the data, supporting data is not available.

## Abbreviations

SFBT: Solution Focused Brief Therapy
L&D: Liaison and Diversion
RCT: Randomised controlled trial
SAU: Services as usual
LSCFT: Lancashire and South Cumbria NHS Foundation Trust
SDQ: Strengths and Difficulties Questionnaire
MCID: Minimally clinically important difference
MDES: Minimally detected effect size
SD: Standard deviation
PNC: Police National Computer
GLM: Generalised linear models
GLMM: Generalised linear mixed model
QQ: Quantile–quantile [plot]
MAR: Missing-at-random
NMAR: Not missing-at-random
IV: Instrumental variable
ICC: Intra-correlation coefficient
SAE: Serious adverse event
AE: Adverse event
PPI: Patient and public involvement
PAG: Project Advisory Group
PI: Principal investigator
