# Correction: Statistical analysis plan for the SOLUTIONS randomised controlled trial with internal pilot: Solution Focused Brief Therapy (SFBT) in 10–17-year-olds presenting at police custody

**DOI:** 10.1186/s13063-024-08529-4

**Published:** 2024-10-13

**Authors:** Paul A. Thompson, Gwenllian Moody, Elinor Coulman, Eleri Owen-Jones, Faizan Patel, Kylie M. Gray, Richard P. Hastings, Andrea Longman, Fiona Lugg-Widger, Jeremy Segrott, Julia Badger, Samantha Flynn, Peter E. Langdon, Rebecca Playle

**Affiliations:** 1 School of Education, Language and Communication Sciences, University of Warwick, Coventry, UK; 2 Centre for Trials Research, Cardif University, Cardif, UK; 3 Department of Education, University of Oxford, Oxford, UK; 4 Centre for Research in Intellectual and Developmental Disabilities (CIDD), University of Warwick, Warwick, CV4 7AL UK

**Correction** **:** **Trials 25, 633 (2024)**

**https://doi.org/10.1186/s13063-024-08457-3**

Following the publication of the original article [1], we were notified of a typo in the title.

Originally published title:

“Statistical analysis plan for the SOLUTIONS randomised controlled trial with internal pilot: Solution Focused Brief Therapy (SFBT) in 10–17-year-olds presenting at policy custody”

Corrected title:

“Statistical analysis plan for the SOLUTIONS randomised controlled trial with internal pilot: Solution Focused Brief Therapy (SFBT) in 10–17-year-olds presenting at police custody”

The original article has been corrected.
