# Supplementary material for: Statistical analysis plan for the SOLUTIONS randomised controlled trial with internal pilot: Solution Focused Brief Therapy (SFBT) in 10–17 year olds presenting at police custody
Source: Trials. 2024 Sep 28;25:633. doi: 10.1186/s13063-024-08457-3 (PMC11438289; doi:10.1186/s13063-024-08457-3)
Supplement: Supplementary file 1 — Supplementary Material 1. [file 13063_2024_8457_MOESM1_ESM.docx]

### Section A: Consort template


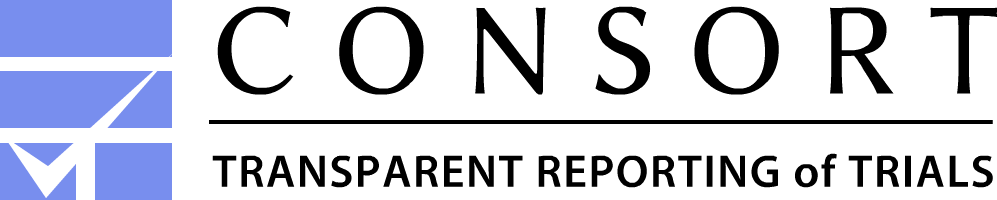


**CONSORT 2010 Flow Diagram**

Analysed (n= )
♦ Excluded from analysis (give reasons) (n= )

Analysed (n= )
♦ Excluded from analysis (give reasons) (n= )

Lost to follow-up (give reasons) (n= )

Discontinued intervention (give reasons) (n= )

Lost to follow-up (give reasons) (n= )

Discontinued intervention (give reasons) (n= )

## Enrollment

Allocated to intervention (n= )

♦ Received allocated intervention (n= )

♦ Did not receive allocated intervention (give reasons) (n= )

Allocated to intervention (n= )

♦ Received allocated intervention (n= )

♦ Did not receive allocated intervention (give reasons) (n= )

Assessed for eligibility (n= )

Excluded (n= )

♦  Not meeting inclusion criteria (n= )

♦  Declined to participate (n= )

♦  Other reasons (n= )

Ranomized (n= )

## Allocation

## Follow-Up

## Analysis

### Section B: Results tables templates

Table B1. Baseline characteristics of trial arms as randomised

| Individual-level | Whole Group | | Intervention group | | Control group | |  |
| --- | --- | --- | --- | --- | --- | --- | --- |
| (categorical) | n/N | Count (%) | n/N | Count (%) | n/N | Count (%) |  |
|  | (missing) |  | (missing) |  | (missing) |  |  |
| Sex (Female) |  |  |  |  |  |  |  |
| **Custody** |  |  |  |  |  |  |  |
| Blackburn |  |  |  |  |  |  |  |
| Preston |  |  |  |  |  |  |  |
| Blackpool |  |  |  |  |  |  |  |
| Barrow |  |  |  |  |  |  |  |
| Lancaster |  |  |  |  |  |  |  |
| ONLINE |  |  |  |  |  |  |  |
| Individual-level | n/N | Mean (SD) | n/N | Mean (SD) | n/N | Mean (SD) | Effect size (g) |
| (continuous) | (missing) |  | (missing) |  | (missing) |  |  |
| Age |  |  |  |  |  |  |  |
| VIQ |  |  |  |  |  |  |  |
| Baseline SRDM |  |  |  |  |  |  |  |
| Baseline GARM |  |  |  |  |  |  |  |
| Baseline Arrests |  |  |  |  |  |  |  |
| Baseline Cautions |  |  |  |  |  |  |  |
| Baseline Reprimands |  |  |  |  |  |  |  |
| Baseline Warnings |  |  |  |  |  |  |  |
| Baseline Convictions |  |  |  |  |  |  |  |

Table B2. Baseline characteristics based on data completeness

| Individual-level | Complete cases | | Lost to follow up | |
| --- | --- | --- | --- | --- |
| (categorical) | n/N | Count (%) | n/N | Count (%) |
|  | (missing) |  | (missing) |  |
| Sex (Female) |  |  |  |  |
| Custody |  |  |  |  |
| Individual-level | n/N | Mean (SD) | n/N | Mean (SD) |
| (continuous) | (missing) |  | (missing) |  |
| Age |  |  |  |  |
| VIQ |  |  |  |  |
| Baseline SRDM |  |  |  |  |
| Baseline GARM |  |  |  |  |
| Baseline Arrests |  |  |  |  |
| Baseline Cautions |  |  |  |  |
| Baseline Reprimands |  |  |  |  |
| Baseline Warnings |  |  |  |  |
| Baseline Convictions |  |  |  |  |

Table B3. Primary analysis model coefficients

|  | **Primary analysis** | |
| --- | --- | --- |
| ***Coefficient*** | *Estimates* | *CI* |
| Intercept |  | [ , ] |
| SRDM Baseline |  | [ , ] |
| Custody suite 2 |  | [ , ] |
| Custody suite 3 |  | [ , ] |
| VIQ |  | [ , ] |
| Age |  | [ , ] |
| Sex |  | [ , ] |
| Trial arm |  | [ , ] |
|  | | |
| Observations |  | |
| R^2^ / adjusted R^2^ | ___ / ____ | |
| ** p<0.05   ** p<0.01   *** p<0.001* | | |

Table B4. Subgroup analysis model coefficients

|  | **LD subgroup analysis** | | **Callous and unemotional traits subgroup analysis** | |
| --- | --- | --- | --- | --- |
| ***Coefficient*** | *Estimates* | *CI* | *Estimates* | *CI* |
| Intercept |  | [ , ] |  | [ , ] |
| SRDM Baseline |  | [ , ] |  | [ , ] |
| Custody suite 2 |  | [ , ] |  | [ , ] |
| Custody suite 3 |  | [ , ] |  | [ , ] |
| VIQ |  | [ , ] |  | [ , ] |
| Age |  | [ , ] |  | [ , ] |
| Sex |  | [ , ] |  | [ , ] |
| Trial arm |  | [ , ] |  | [ , ] |
| LD x Trial Arm |  | [ , ] |  |  |
| CALLOUS x Trial  Arm |  |  |  | [ , ] |
|  | | |  |  |
| Observations |  | |  |  |
| R^2^ / adjusted R^2^ | ___ / ____ | |  |  |
| ** p<0.05   ** p<0.01   *** p<0.001* | | |  |  |

Table B5. Two level model for additional analysis allowing for clustering of therapists in intervention arm.

|  | **Primary analysis(two-level)** | |
| --- | --- | --- |
| ***Coefficient*** | *Estimates* | *CI* |
| Intercept |  |  |
| SRDM Baseline |  |  |
| Custody suite 2 |  |  |
| Custody suite 2 |  |  |
| VIQ |  |  |
| Age |  |  |
| Sex |  |  |
| Trial Arm |  |  |
| **Random Effects** | | |
| σ^2^ |  | |
| τ_00_ | ____therapist_ID_ | |
| ICC |  | |
| N | __ _therapist_ID_ | |
| Observations |  | |
| Marginal R^2^ / Conditional R^2^ | ___ / ____ | |
| ** p<0.05   ** p<0.01   *** p<0.001* | | |

| **Outcome Measures** | **Baseline** | | | | **6 month follow-up** | | | | **^a^Unadjusted mean difference** | **^b^Adjusted mean difference (covariates)** | **^c^Adjusted mean difference (therapist clustering)** |
| --- | --- | --- | --- | --- | --- | --- | --- | --- | --- | --- | --- |
|  | **SAU** | | **SAU + Intervention** | | **SAU** | | **SAU + Intervention** | |  |  |  |
|  | **n** | **Mean (SD)** | **n** | **Mean (SD)** | **n** | **Mean (SD)** | **n** | **Mean (SD)** | **Difference (95% CI), p value** | **Difference (95% CI), p value** | **Difference (95% CI), p value** |
| SRDM |  |  |  |  |  |  |  |  |  |  |  |
| GARM |  |  |  |  |  |  |  |  |  |  |  |
| **Criminal Offences** |  |  |  |  |  |  |  |  |  |  |  |
| Arrests |  |  |  |  |  |  |  |  |  |  |  |
| Cautions |  |  |  |  |  |  |  |  |  |  |  |
| Reprimands |  |  |  |  |  |  |  |  |  |  |  |
| Warnings |  |  |  |  |  |  |  |  |  |  |  |
| Convictions |  |  |  |  |  |  |  |  |  |  |  |
| ^a^single-level model adjusted for baseline score, custody suite, and VIQ. | | | | | | |  |  |  |  |  |
| ^b^Single level model adjusting for sex, age, VIQ, custody suite, and baseline score | | | |  |  |  |  |  |  |  |  |
| ^c^Multilevel model adjusted for therapist clustering (intervention arm only), VIQ, custody suite, and baseline score | | | | | |  |  |  |  |  |  |
| ** p<0.05   ** p<0.01   *** p<0.001* |  |  |  |  |  |  |  |  |  |  |  |

Table B6. Primary analyses summary table.

Table B7. Missing data analysis using imputed data sets.

| **Outcome Measures** | **Baseline** | | | | **6 month follow-up** | | | | **^a^Unadjusted mean difference** |
| --- | --- | --- | --- | --- | --- | --- | --- | --- | --- |
|  | **SAU** | | **SAU + Intervention** | | **SAU** | | **SAU + Intervention** | |  |
|  | **n** | **Mean (SD)** | **n** | **Mean (SD)** | **n** | **Mean (SD)** | **n** | **Mean (SD)** | **Difference (95% CI), p value** |
| SRDM |  |  |  |  |  |  |  |  |  |
| GARM |  |  |  |  |  |  |  |  |  |
| **Criminal Offences** |  |  |  |  |  |  |  |  |  |
| Arrests |  |  |  |  |  |  |  |  |  |
| Cautions |  |  |  |  |  |  |  |  |  |
| Reprimands |  |  |  |  |  |  |  |  |  |
| Warnings |  |  |  |  |  |  |  |  |  |
| Convictions |  |  |  |  |  |  |  |  |  |
| ^a^single-level model adjusted for baseline score, custody suite, and VIQ. | | | | | | |  |  |  |
| ** p<0.05   ** p<0.01   *** p<0.001* |  |  |  |  |  |  |  |  |  |

Table B8. Instrumental variable analysis for fidelity and adherence

| **Coefficient** | **Primary** | | **IV analysis (fidelity)** | | **IV analysis (Adherence)** | |
| --- | --- | --- | --- | --- | --- | --- |
|  | **Estimates** | **CI** | **Estimates** | **CI** | **Estimates** | **CI** |
| Intercept |  | [ , ] |  | [ , ] |  | [ , ] |
| Baseline SRDM |  | [ , ] |  | [ , ] |  | [ , ] |
| Custody suite 2 |  | [ , ] |  | [ , ] |  | [ , ] |
| Custody suite 3 |  | [ , ] |  | [ , ] |  | [ , ] |
| Trial Arm |  | [ , ] |  | [ , ] |  | [ , ] |
| VIQ |  | [ , ] |  | [ , ] |  | [ , ] |
| Age |  | [ , ] |  | [ , ] |  | [ , ] |
| Sex |  | [ , ] |  | [ , ] |  | [ , ] |
| **observations** |  |  |  |  |  |  |
| **R^2^ / Adjusted R^2^** |  |  |  |  |  |  |
| ** p<0.05   ** p<0.01   *** p<0.001* |  |  |  |  |  |  |

### Section C: Progression criteria

**Recruitment**

(i) Up to 50% of overall target (n=222) (CYP) within first 7 months of recruitment to the trial

(green=80 to 100%; amber=60 to 79%; red=<60%);

1400 children have been referred to Liaison and Diversion across LSCFT’s x 3 custody suites

between 1st April 2021 and 31st May 2022. Local data indicates numbers are increasing.

Liaison and Diversion as a service, have high engagement rates with children and young

people. Around 80% of children referred to the liaison and diversion service, accept the

offer of an assessment.

**Randomisation**

(i) Number of CYP randomised (of CYP consented green=≥90%; amber=50-89%; red=<49%).

Retention

(i) Number of CYP (of randomised) not explicitly withdrawn from the trial (at 6-months:

green=≥80%; amber=50-79%; red=<50%);

(ii) Are the approaches to maximise retention acceptable to participants in this trial?

(assessed qualitatively through interviews with a small sample of CYP and

parents/guardians).

**Fidelity and adherence**

(i) Fidelity assessed according to a fidelity checklist (developed in collaboration with the

delivery team, prior to the internal pilot – see later in Process Evaluation) (green=≥80 of

sessions meet criteria; amber=50-79%; red=<50%);

(ii) Adherence: session attendance (green=≥ 66.6% of scheduled sessions attended; amber=

50-66.6%; red=<50%) (an average of the number of sessions)

**Outcomes**

(i) Willingness of CYP to participate in trial processes (data completeness for 6-month Self

Report Delinquency Measure: green=≥75%; amber=50-74%; red=<50%).

**How does SFBT differ from SAU?**

(i) SAU data from intervention and control groups in the internal pilot will be examined for

any overlap with the content of the SFBT intervention (assessed by SAU questions in

baseline questionnaire, and qualitatively through interviews with a small sample of CYP and

parents/guardians), and to;

(ii) examine whether SAU is similar in the intervention and control groups, with the data

collated from services received (assessed by SAU questions in questionnaires, and

qualitatively through interviews with CYP, parents/guardians, and practitioners)
